# Estimating the Current Routes of Transmission in HIV-1 F1 Subtype Infected Persons in Romania: Differences Between Self-Reporting and Phylogenetic Data

**DOI:** 10.3390/pathogens13110960

**Published:** 2024-11-04

**Authors:** Robert Hohan, Simona Paraschiv, Ionelia Nicolae, Dan Oțelea

**Affiliations:** 1 National Institute for Infectious Diseases “Prof. Dr. Matei Bals”, 021105 Bucharest, Romania; hohan.robert@gmail.com (R.H.); batan_ioana@yahoo.com (I.N.); dotelea@mateibals.ro (D.O.); 2 Virology Department, University of Medicine and Pharmacy “Carol Davila”, 050474 Bucharest, Romania

**Keywords:** HIV, transmission, phylogenetic, clusters, F1, Romania, MSM

## Abstract

Monitoring the HIV epidemic in Romania has proven challenging due to many factors, including the reluctance of newly diagnosed patients to disclose relevant epidemiological aspects during the clinical interview, such as sexual orientation or the existence of previous issues with injectable drug usage. We propose in this study a molecular approach to mitigate this problem with the help of bioinformatic tools, such as cluster analysis of phylogenetic trees. Both a maximum likelihood estimation, as implemented with FastTree, and a Bayesian approach, as used in BEAST, have been applied to our data set of 312 HIV subtype F1 *pol* gene sequences. ClusterPicker was used in order to identify groups of sequences and indicate similarities possibly related to the route of transmission. An important observation from this analysis is that transmission between men who have sex with men (MSM) is likely occurring in networks significantly larger than previously assessed by self-reported data (65% from the phylogenetic tree versus 37% from self-declared affiliation). Cluster analysis can help identify risk factors, reveal transmission trends, and, consequently, advise prevention programs.

## 1. Introduction

Due to the rapid development of computational methods applied to the study of microorganisms, phylogenetic analysis has contributed to an increasing number of different research topics, such as characterizing epidemic dynamics, detecting migration patterns, reconstructing transmission networks in high-risk populations, identifying genes that are under selective pressure, estimating the age of particular pathogens, and many other applications [1,2,3,4]. HIV is one of the most studied viruses, not least because of the high mutation rate that makes it a candidate for phylogenetic analysis. Romania is performing continuous national surveillance of new HIV-1 cases through the Department for Monitoring and Evaluation of HIV/AIDS Infection in Romania, formerly known as the National Commission for the Fight Against AIDS (CNLAS) [5]. However, the corresponding genotypic data are not systematically generated due to financial limitations.

Transmission trends have varied over time in Romania [4,6,7]. A major event in the late ‘80s and early ‘90s was a high number of nosocomial infections in the pediatric population. The epidemics started with the F1 subtype circulating in this particular population but also in adults [6], where heterosexual contact was initially responsible for HIV transmission. However, ten years ago, an outbreak started in people who inject drugs (PWID) [8]. Furthermore, according to the national data, there has been a steady increase in the number of HIV cases among men having sex with men (MSM), rising from about 10% of the new reported cases in 2013 to 29% in 2023 [9].

HIV sequences generated when testing to detect transmitted drug resistance mutations are also useful for subtyping purposes and for identifying transmission clusters in newly diagnosed cases [10]. Cluster analysis can help identify risk factors, reveal transmission trends, and, consequently, advise prevention programs. Clusters in epidemiology are generally characterized as an atypical concentration of infections that is considered to exceed what would be anticipated by random transmission. Within a phylogenetic tree, clusters comprise sequences from various patients that share a recent common ancestor. These clusters are represented as distinct groupings where there is a high level of confidence, indicating a likelihood of recent or ongoing transmission [11].

The aim of the present study was to perform cluster analysis on a large F1 subtype HIV dataset consisting of pol sequences from newly diagnosed patients in the last decade and see whether there is a correlation between the declared route of transmission and the sequence clustering.

## 2. Materials and Methods

### 2.1. Study Population

We included in our analysis 312 HIV-1 F1 subtype nucleotide sequences from infected patients who were diagnosed between 2019 and 2022. This F1 subtype dataset is representative of Romania in terms of geographic origin, age distribution, and proportions of the self-reported route of transmission. The average age at diagnosis for this study was 36.48, whereas the national reports indicate that 70% of newly diagnosed persons for this period were between 30 and 49 years old [9]. The gender ratio for this study was 80% male, similar to national reports (78%). The samples analyzed in the study were collected from the main population centers of Romania. All the available sequence information at the national level is collected by the Molecular Diagnostics Laboratory from the National Institute for Infectious Diseases “Prof. Dr. Matei Bals”, the reference center for HIV-1 genotyping resistance testing. Another 195 sequences, also belonging to the F1 subtype, that were generated and reported circulating in newly diagnosed patients from 2003–2016, were also included in the analysis. These last sequences correspond to MSM, PWID, and sexual transmissions. As references, we used F1 subtype sequences from Romanian persons nosocomially infected in infancy (*n* = 16).

The sequences were generated using the ViroSeq HIV-1 Genotyping System (Celera Diagnostics, Alameda, CA, USA) and covered the complete protease (PR) gene and part (the first 1004 nucleotides) of the reverse transcriptase (RT) gene. Both the REGA version 3.0 algorithm and the COMET version 2.4 tools were used for subtype assignment [12,13]. Both tools returned similar results.

Clinical and epidemiological data were collected from these patients. The study was conducted according to the Declaration of Helsinki and approved by the Ethical Committee of the National Institute for Infectious Diseases ‘Prof. Dr. Matei Bals’ (approval C09808/2024). Furthermore, after the initial selection, the patient names were anonymized, and no identifiable data remained in the submitted dataset.

### 2.2. Cluster Analysis

The reference sequences were selected from the HIV-1 Los Alamos Database and GenBank [14,15]. The most similar HIV sequences were retrieved using BLAST, and duplicates were subsequently deleted. The final dataset consisted of 560 sequences, with the resulting alignment being 1301 nucleotides long. For the outgroup, we used 18 sequences of subtype B. Details about the final dataset, including references and outgroup sequences, are presented in Supplementary Table S1.

Analysis was performed by constructing maximum likelihood (ML) phylogenetic trees using FastTree 2 software [16], taking into account both the General Time-Reversible model and the gamma model with 20 rate categories.

In order to find significant clusters within the phylogenetic tree, an automated analysis tool, ClusterPicker version 1.2.3 (CP), was used, with thresholds of at least 90% bootstrap support and a genetic distance below 10%. In order to infer the most likely route of transmission for these infections, the identified clusters were then analyzed in the context of the epidemiological self-reporting data. This analysis and the subsequent visualizations were performed with the aid of the FigTree version 1.4.4 [17].

The clusters identified by the ML phylogenetic tree (SH-like support higher or equal to 0.9) were further confirmed by performing Bayesian phylogenetic analysis. Bayesian phylogenetic analysis was conducted using BEAST version 2.5 using a Bayesian Skyline coalescent tree prior and the uncorrelated lognormal relaxed clock model [18,19,20]. Two Markov Chain Monte Carlo (MCMC) runs were computed separately for 10^7^ generations with a burn-in of 10%. The output of the MCMC analysis was tested for convergence by means of effective sampling size (ESS > 200) using the program Tracer v1.7 [21]. TreeAnnotator program was used to generate the maximum clade credibility (MCC) [22].

### 2.3. Statistical Analysis

The resulting data regarding the route of transmission were analyzed using a chi-squared test in order to test the null hypothesis that there are no differences between the self-reported information and what the cluster analysis indicated.

## 3. Results

A total of 560 sequences were aligned, consisting of 18 as the outgroup (subtype B), 16 from the Romanian cohort of pediatric infections (diagnosed in 2003–2004), 35 F1 reference sequences retrieved by BLAST, 179 covering a period from 2007 to 2016 (with available epidemiological data and self-reported route of transmission), and the 312 F1-subtype HIV sequences to be analyzed. Among our dataset, 236 (75.6%) had a presumptive cause of infection, as recorded during the clinical interview at the moment of diagnosis. However, due to the stigma associated with either a more risk-tolerant sexual lifestyle or difficulty in admitting to a drug abuse issue, the real mode of transmission may not always be declared truthfully. Therefore, we used different bioinformatics tools based on distinct phylogenetic approaches (maximum-likelihood and Bayesian) in order to identify clusters of transmission among newly diagnosed Romanian patients infected with F1 subtype strains.

After constructing a phylogenetic tree with FastTree, the ClusterPicker algorithm indicated 25 clusters with 3 or more branches. Groups of two sequences (pairs) were not further evaluated. The resulting clusters were also tested through a Bayesian statistics method for confirmation. The identified transmission clusters are summarized in Table 1.

The confirmed clusters were analyzed in the context of the epidemiological information available for the sequences collected previously in 2003–2016 and taking into account self-reported transmission data for the patients diagnosed in 2019–2022. Several categories of transmission clusters were observed:

- Seven clusters (comprising in total of 62 sequences, 37 from 2019 to 2022) of strains associated with heterosexual transmission;
- Nine clusters (271 in total, of which 204 sequences were from 2019 to 2022) of strains associated with MSM transmission;
- Nine clusters (89 sequences, of which 35 sequences were from 2019 to 2022) of strains affiliated with people who inject drugs (PWID).

Using this approach, 276 (88%) sequences from patients with subtype F1 HIV-1 infection and diagnosed during 2019–2022 were included in clusters defined by a consensus sequence usually (but not exclusively) associated with a route of transmission.

**Figure 1:**
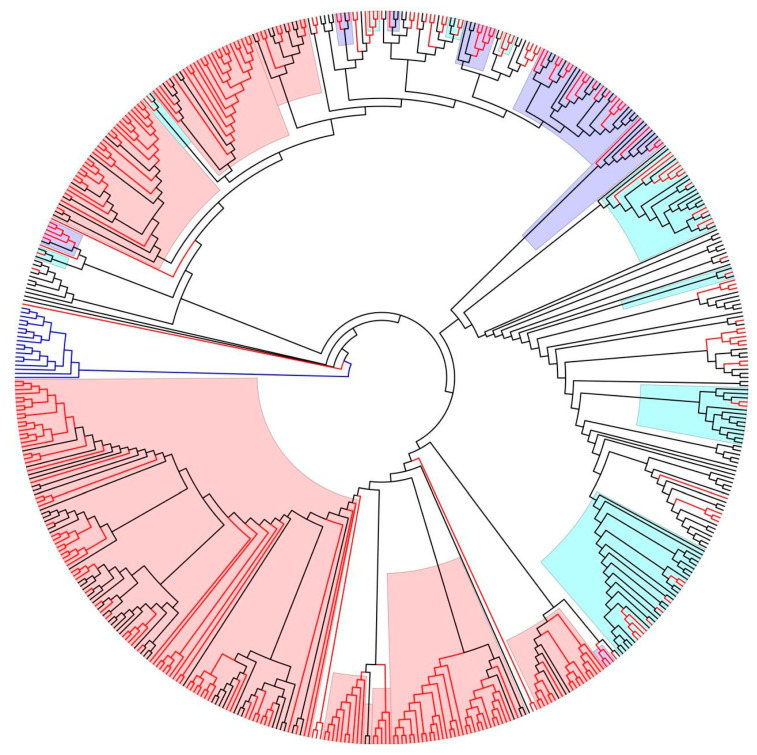
Radial representation of the phylogenetic ML tree: black branches represent F1 reference sequences (retrieved by BLAST plus other Romanian F1 sequences collected previously (2003–2016), blue branches represent the subtype B outgroup sequences, and red branches represent the F1 sequences from newly diagnosed patients between 2019 and 2022. Highlighting is as follows: light red represents MSM-associated clusters, lavender blue represents heterosexual-associated clusters, and cyan is for transmission clusters correlated with PWID.

A visual representation of these results can be seen in Figure 1, which shows the phylogeny with the relevant clusters highlighted by route of transmission. The largest transmission cluster identified was associated with MSM transmission and presented 122 sequences, of which 79 sequences (64.7%) corresponded to patients recently diagnosed between 2019 and 2022. Another large cluster was associated with PWID and consisted of 33 sequences, but only 10 (30%) were from patients diagnosed in recent years. The majority of sequences from this cluster corresponded to PWID diagnosed with HIV-1 in 2011–2016.

**Figure 2:**
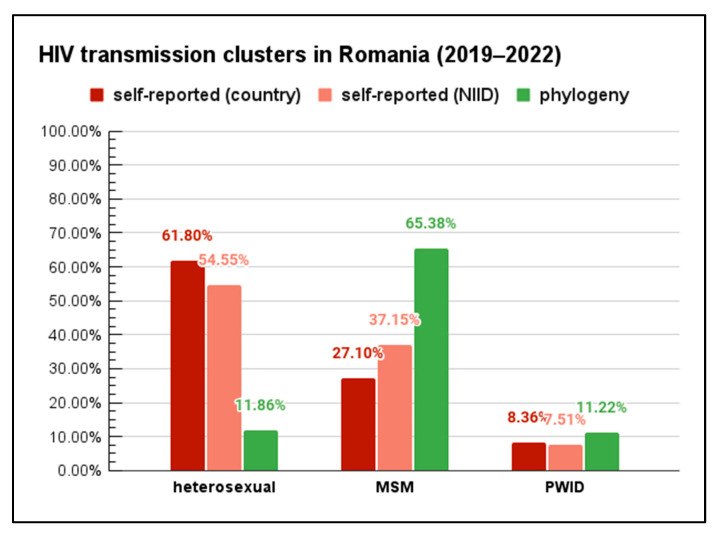
Proportion of new diagnoses with a self-reported putative route of transmission or associated with a specific transmission cluster from the phylogenetic analysis. MSM—men having sex with men. PWID—people who inject drugs. NIID—National Institute for Infectious Diseases “Prof. Dr. Matei Bals”.

By comparing the transmission route for the analyzed dataset with the national data provided by National Committee for fighting against AIDS/HIV in Romania (CNLAS) for the same period of time, it can be observed that the proportions are relatively similar to what the patients themselves self-reported (Figure 2). However, when taking into account the clustering analysis, there are differences in this distribution. According to this analysis, the sequences associated with the MSM route are significantly higher in number compared with those from self-reported data: χ2 (DF = 4, N = 3272) = 279.0198, *p* < 0.00001. Differences appear also for the heterosexual route of transmission but in the opposite sense.

## 4. Discussion

The objective of ending the transmission of HIV by 2030 has many prerequisites [23,24]. These include rapid access to treatment of all infected people, widespread access to diagnosis, efficient monitoring of the infected patients, and prevention of transmission. There are several means of implementing the latter objective (pre- and post-exposure prophylaxis, advocacy for condom use, and achieving undetectable status in discordant couples), but at the population level, identifying the groups at risk and designing policies that can reach these groups is paramount and often challenging. Several barriers hamper these goals, and among them, cultural differences and stigma are still an issue. For instance, self-declared sexual orientation and usage of injectable drugs often do not accurately reflect the epidemiological situation. Our study explored the possibility of using a molecular analysis to improve our estimation of the size of such populations of interest.

Molecular phylogeny approaches have proven over time their ability to add important insights into particular epidemics. The viral sequences harbor evolutionary markers that can be used to estimate the age of a viral strain to identify networks and patterns of transmission [25,26].

Several factors may influence the phylogenetic results and thus the identification of transmission clusters. The broader definition of a phylogenetic cluster is a group of viral sequences from different individuals that share a higher similarity than sequences from other groups and have a common ancestor [27]. Clusters are usually technically defined by high branch support (bootstrap, approximate likelihood ratio test, posterior probability) and low genetic or patristic distance [28,29]. There are currently debates around the methodology used to identify phylogenetic clusters, the threshold cut-offs, and the genomic regions that are most appropriate for HIV-1 analysis. There is still a consensus though that a very important aspect when interpreting phylogenetic data is the sample size and density. The methodology used in this study included all of the sequences generated from newly diagnosed patients (2019–2022) infected with the F1 subtype and took into consideration both high branch support (SH-like values generated in FastTree and posterior probabilities from Bayesian analysis) and maximum cluster genetic distance, as implemented in ClusterPicker [11], one of the most cited software programs developed for identifying transmission clusters [1,26]. The genomic region analyzed (partial *pol* gene) is routinely used for detecting resistance mutations but also provides a good phylogenetic signal. Our analysis was performed on sequences collected at the national level with a limited sampling window, increasing the chances of identifying recent ongoing transmission networks.

Similar approaches were used to characterize viral transmission (HCV, West Nile virus, SARS-CoV-2, influenza virus, bluetongue virus, etc.) within different populations, but the studies performed on HIV-1 have been those that benefit most from such tools [30]. A decade ago, Greece was highly affected by a sub-epidemic among intravenous drug users, and the prompt signaling of this problem together with molecular characterization of this epidemic using phylogeny helped adjust prevention programs [31,32]. Phylogenetic analysis was also used in criminal courts [33] or to investigate nosocomial transmission [34].

In line with our study, Otani and colleagues recently published a paper on CRF01_AE cases in Japan showing that MSM were five times more likely to be in a transmission cluster compared with heterosexuals and were the main contributors to a transmission chain [35]. Heterosexual transmission is more often associated with single introduction events or limited clustering due to sampling (most of them are late presenters), but it is also associated with social, behavioral, and cultural factors.

The discrepancy between self-reported data on the possible cause of infection and the results obtained from phylogenetic analysis strengthens the idea that stigma associated with a more diverse and/or risk-tolerant sexual lifestyle is still present in Romanian society. While a clearcut categorization of the HIV-1 transmission route cannot be inferred just based on this type of approach, there is reason to believe that some types of transmission are more prevalent than usually reported in MSM and in other populations of interest, such as PWID.

As stated above, it is quite obvious that sequences per se, even when they show a tendency to cluster, can offer only limited information about the actual route of transmission; similar viral variants can be transmitted via different types of events, and some people can be associated with more than one possible route (e.g., PWID and MSM, PWID and heterosexuals). In Romania, self-reported MSM transmission registered a continuous increase in the last decade, currently accounting for about 30% of newly diagnosed cases [9]. Mainly, it was HIV-1 subtype B that was formerly associated with this type of transmission [4], but our data show that a large proportion of recently diagnosed patients, self-declared as MSM, are infected with the F1 subtype.

In addition, the data suggest that the tendency is still to underreport certain types of transmission (MSM, PWID), and therefore, there is a need to better define the populations at risk in order to reach out and develop efficient public health policies.

## 5. Conclusions

Self-reported routes of transmission may not accurately describe populations at risk of acquiring HIV (e.g., MSM, PWID), and molecular analysis can be an important tool that helps improve this estimation. Prevention programs targeting risk populations in Romania should be considered.

## Figures and Tables

**Table 1:** Summary of all the F1 subtype clusters generated with ClusterPicker software, as it was applied to the phylogenetic tree generated with FastTree. MSM—men having sex with men. PWID—people who inject drugs.

Cluster ID	Total Sequences (N)	Newly Diagnosed (N)	Sequences Most Often/Exclusively Encountered in
1	29	16	heterosexuals
2	8	5	heterosexuals
3	6	5	heterosexuals
4	9	4	heterosexuals
5	4	3	heterosexuals
6	3	3	heterosexuals
7	3	1	heterosexuals
8	122	79	MSM
9	34	31	MSM
10	36	29	MSM
11	28	24	MSM
12	19	17	MSM
13	10	10	MSM
14	14	8	MSM
15	5	5	MSM
16	3	1	MSM
17	23	12	PWID
18	33	10	PWID
19	3	3	PWID
20	14	2	PWID
21	4	2	PWID
22	3	2	PWID
23	3	2	PWID
24	3	1	PWID
25	3	1	PWID

## Data Availability

The sequences analyzed in this study were submitted to NCBI GenBank and have the following accession numbers PQ512891-PQ513202. Details about the reference sequences used in this analysis can be found in Supplementary Table S1 (https://www.mdpi.com/article/10.3390/pathogens13110960/s1, Table S1: Final dataset: F1 subtype sequences analyzed in this study (2019–2022), references and outgroup sequences).

## Supplementary Materials

The following supporting information can be downloaded at:https://www.mdpi.com/article/10.3390/pathogens13110960/s1, Table S1: Final dataset: F1 subtype sequences analyzed in this study (2019–2022), references and outgroup sequences
