# Supplementary material for: Estimating the Current Routes of Transmission in HIV-1 F1 Subtype Infected Persons in Romania: Differences Between Self-Reporting and Phylogenetic Data
Source: Pathogens. 2024 Nov 4;13(11):960. doi: 10.3390/pathogens13110960 (PMC11597275; doi:10.3390/pathogens13110960)
Supplement: Supplementary file 1 [file pathogens-13-00960-s001.zip › pathogens-3272366-supplementary.pdf]

description	newly diagnosed	PWID	MSM	pediatric/nosocomial	heterosexual	BLAST	outgroup
subtype	F1	F1	F1	F1	F1	F1	B
period	2019-2022	2011-2016	2007-2014	2003-2004	2007-2008	1999-2020	2009-2016
N	312	146	21	16	12	35	18
	GenBank accession numbers						
		KJ194673		HM191559	JQ083014	AJ249238	JN982133
	PQ512891	KJ194674	PQ513203	HM191560	JQ083018	AJ287042	JN982136
	-	KJ194675	-	HM191561	JQ083025	AY173957	JN982139
	PQ513202	KJ194676	PQ513223	HM191562	JQ083026	AY455782	JN982143
		KJ194677		HM191564	JQ083041	FJ209060	JN982144
		KJ194678		HM191565	JQ083044	FJ405153	JN982145
		KJ194681		HM191566	JQ083048	FJ481661	JN982146
		KJ194682		HM191567	JQ083051	FJ900268	JN982154
		KJ194684		JQ082984	JQ083056	GQ462360	JN982165
		KJ194685		JQ082985	JQ083062	KM284389	JN982174
		KJ194686		JQ082987	JQ083067	KY392770	KJ194727
		KJ194687		JQ082988	JQ083074	LC766260	KJ194730
		KJ194691		JQ082989		MF403302	KJ194731
		KJ194692		JQ082990		MF403306	KJ194732
		KJ194693		JQ082999		MF403319	KX159008
		KJ194695		JQ083001		MG365771	KX159042
		KJ194696				MK177663	KX159048
		KJ194699				MK177687	OM857444
		KJ194700				MK177746	
		KJ194701				MK177773	
		KJ194702				MN133179	
		KJ194703				MN178953	
		KJ194705				MT222953	
		KJ194707				MT417769	
		KJ194708				MT570389	
		KJ194709				MT570393	
		KJ194710				MT570419	
		KJ194711				MT570445	
		KJ194712				MT570478	
		KJ194713				MT570543	
		KJ194714				MT570661	
		KJ194717				MT570670	
		KJ194718				MT571157	
		KJ194719				ON703535	
		KJ194720				ON989250	
		KJ194735					
		KJ194736					
		KJ194737					
		KJ194739					
		KJ194740					
		KJ194742					
		KJ194743					
		KJ194744					
		KJ194745					
		KJ194747					
		KJ194749					
		KJ194750					
		KJ194751					
		KJ194752					
		KJ194753					
		KJ194754					
		KJ194755					
		KJ194756					

KJ194757
KJ194758
KJ194759
KJ194760
KJ194762
KJ194763
KJ194764
KJ194765
KJ194767
KJ194768
KJ194769
KJ194770
KJ194771
KJ194772
KJ194773
KJ194774
KJ194775
KJ194776
KJ194778
KJ194780
KJ194781
KJ194783
KJ194784
KJ194785
KJ194787
KJ194788
KJ194789
KJ194791
KJ194793
KJ194794
KJ194796
KJ194798
KJ194799
KJ194800
KJ194801
KJ194802
KJ194806
KJ194807
KJ194808
KJ194810
KJ194811
KJ194812
KJ194813
KJ194815
KJ194816
KJ194819
KJ194820
KJ194821
KJ194822
KJ194825
KJ194826
KJ194829
KJ194830
KJ194831
KX158994
KX158996
KX158997
KX158998

KX159000
KX159001
KX159002
KX159005
KX159007
KX159009
KX159010
KX159011
KX159015
KX159018
KX159020
KX159022
KX159023
KX159024
KX159032
KX159035
KX159036
KX159038
KX159040
KX159043
KX159045
KX159047
KX159050
KX159051
KX159053
KX159056
KX159060
KX159062
KX159063
KX159065
KX159066
KX159075
KX159076
KX159077
KX159079
